# Temporary Tumor Shrinkage Following Enfortumab Vedotin Therapy for Metastatic Urothelial Carcinoma After Radical Cystectomy With Neoadjuvant Chemotherapy: A Case Report

**DOI:** 10.7759/cureus.42954

**Published:** 2023-08-04

**Authors:** Sanae Namiki, Daiki Kato, Koji Iinuma, Keita Nakane, Takuya Koie

**Affiliations:** 1 Urology, Gifu University Graduate School of Medicine, Gifu, JPN; 2 Urology, Gifu University, Gifu, JPN

**Keywords:** radical cystectomy, temporary complete response, enfortumab vedotin, metastatic urothelial carcinoma, muscle-invasive bladder cancer

## Abstract

A 39-year-old Japanese male patient presented with a chief complaint of gross hematuria persistent for two months. However, no relevant findings in the patient’s medical and family history were observed. He was diagnosed with muscle-invasive bladder cancer, clinical stage T2bN0M0. After four courses of neoadjuvant chemotherapy with gemcitabine and cisplatin, the tumor size reduced by approximately 30%. The patient underwent robot-assisted radical cystectomy with standard lymph node dissection followed by intracorporeal ileal conduit reconstruction. Histologically, the tumor was diagnosed as a high-grade urothelial carcinoma invading the fatty tissue surrounding the bladder and metastasizing to the lymph nodes, with a pathological stage of ypT3aypN2M0. Four months after surgery, multiple metastases were detected, and treatment with pembrolizumab was initiated immediately. However, the patient did not respond to pembrolizumab. Therefore, a third-line treatment with enfortumab vedotin (EV) was initiated. Thereafter, the metastatic lesion shrank quickly, and the metastatic lesions almost disappeared after two courses of EV administration. Although new metastases were observed at other sites, there has been no regrowth to date. EV-related adverse events were not observed during follow-up. Eighteen months after the surgery, the patient remains alive with multiple metastases. Therefore, the sequence of treatment should be considered to maximize the therapeutic effect of EV, and, consequently, administering EV as early as possible may be important.

## Introduction

Advanced or metastatic urothelial carcinoma (mUC) is widely known as one of the malignant neoplasms that usually advances rapidly and is difficult to cure [1]. Regardless of the response to the platinum-based chemotherapy and subsequent immune checkpoint inhibitors (ICIs), disease progression is often observed in most patients with mUC [1]. Therefore, systemic therapies as third-line treatments have been considered necessary to improve disease control and oncologic outcomes, especially overall survival (OS) [1]. However, until now, approximately two-thirds of the patients have failed to receive further treatment due to tumor progression or worsening of their general condition [2]. With the results of the randomized phase III EV-301 trial, enfortumab vedotin (EV) was found to be effective as a treatment option for patients with treatment-refractory mUC [3]. Therefore, developing a treatment strategy that includes early administration of EV as a third-line treatment is necessary. Based on the results of this trial, EV was approved by the U.S. Food and Drug Administration in 2021 for patients with advanced or mUC whose disease has progressed after the platinum-based chemotherapy followed by ICIs [3]. In this case report, we describe the case of a patient who underwent radical cystectomy after neoadjuvant chemotherapy (NAC), developed early postoperative recurrence, and was treated with EV as the third-line therapy and even achieved disease control at the metastatic sites.

## Case presentation

Patient history and diagnostic evaluation

**Figure 1:**
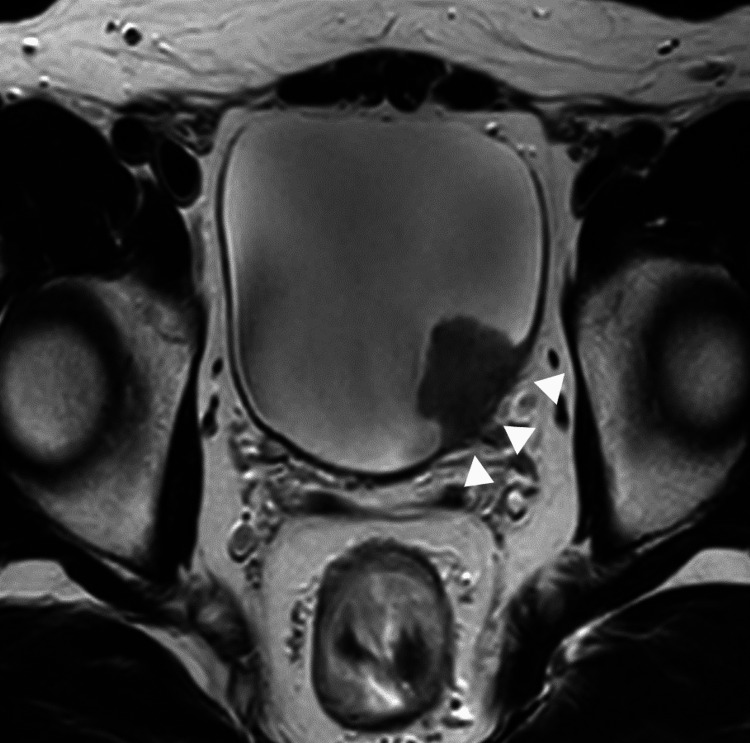
Bladder tumor before treatment. Magnetic resonance image displays multiple bladder tumors with invasion of the muscle layer (white arrowhead).

A 39-year-old Japanese male patient presented with a chief complaint of gross hematuria persistent for two months. However, no relevant findings in the patient’s medical and family history were observed. Ultrasonography and cystoscopy revealed multiple papillary sessile bladder tumors scattered throughout the bladder, with the main lesion being a 26 mm lesion on the left lateral wall. The patient was referred to our institution for further examination. Although there was no evidence of lymph node involvement, pulmonary embolism, or metastasis to other organs on computed tomography (CT), the bladder tumor was strongly suspected to have invaded the muscle layer on magnetic resonance imaging (Figure 1). The patient underwent transurethral resection and biopsy of the bladder tumor, and histology revealed high-grade urothelial carcinoma. The patient was diagnosed with muscle-invasive bladder cancer with clinical stage T2bN0M0.

Clinical course of first-line treatment

**Figure 2:**
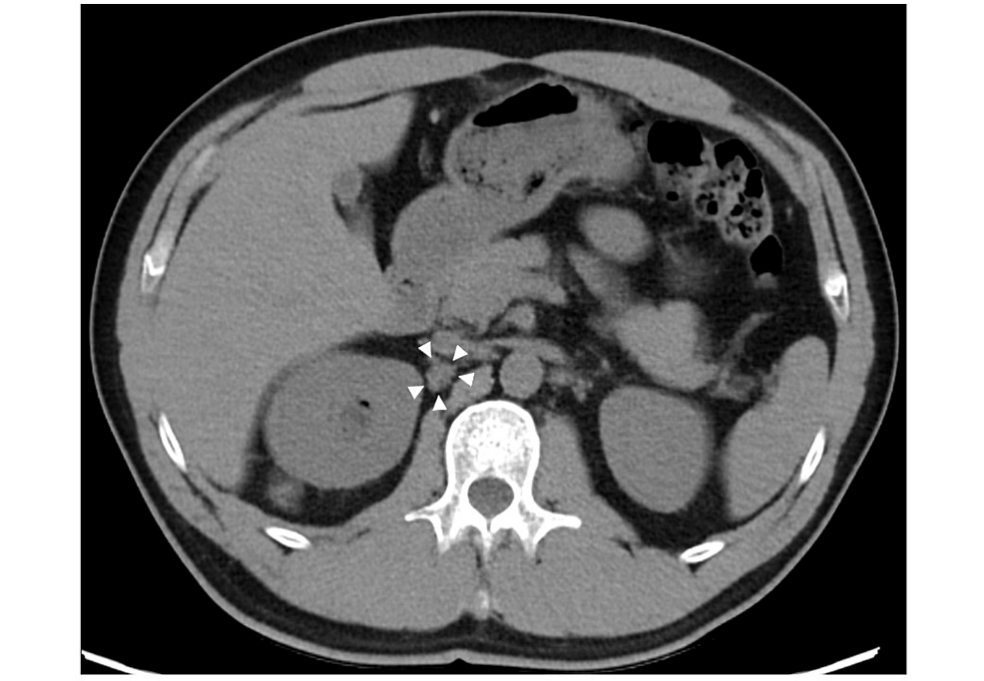
Possibly enlarged lymph node near the right renal hilum. A computed tomography scan performed three weeks after surgery displays a 5 mm, possibly enlarged lymph node near the right renal hilum (white arrowhead).

The patient received four courses of NAC with intravenous gemcitabine and cisplatin (1,000 mg/m^2^ of gemcitabine on days one, eight, and 15 and 35 mg/m^2^ of cisplatin on days one and eight). After NAC, the tumor shrank by approximately 30%, and the patient had a good performance status (PS) of 0. Therefore, robot-assisted radical cystectomy with standard pelvic lymph node dissection (PLND), followed by intracorporeal ileal conduit reconstruction, was performed. Multiple bladder tumors were observed in the surgical specimen, and several tumors extended into the fatty tissues surrounding the urinary bladder. The histological diagnosis was invasive urothelial carcinoma with pathological T3a, lymphovascular invasion, and negative resection margins. In addition, 16 lymph nodes were removed by standard PLND, and tumor metastasis was detected in four of them. The final pathological diagnosis was ypT3aypN2M0. Immunohistochemical staining was not performed in this case. Although the CT scan performed three weeks after surgery revealed a 5 mm possibly enlarged lymph node near the right renal hilum, a definitive diagnosis was not made because of the small size of the lymph node (Figure 2). The patient declined any adjuvant therapy, including ICI. Due to the possibility of early recurrence, a follow-up CT scan was scheduled every two months.

Clinical course of second-line treatment

**Figure 3:**
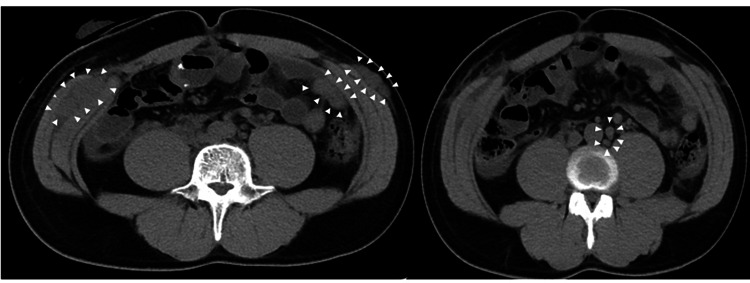
Multiple metastases after the surgery. A computed tomography scan performed four months after the surgery exhibits multiple metastases involving the abdominal wall and para-aortic lymph nodes (white arrowhead).

At four months postoperatively, the patient presented to the emergency department with persistent right lumbar pain. The serum creatinine level (Cr) rapidly worsened from 0.99 mg/dL to 2.75 mg/dL, and the estimated glomerular filtration rate (eGFR) was 21.6 mL/minute/1.73 m^2^. Therefore, contrast-enhanced CT could not be performed. CT scan showed peritoneal dissemination of the bladder cancer, multiple enlarged para-aortic lymph nodes, abdominal wall metastases, and bilateral grade 2 hydronephrosis (Figure 3). In addition, a right nephrostomy was created to aid renal function recovery. Follow-up CT examinations were performed only with plain CT to preserve renal function. One week after emergency hospitalization, the patient was administered pembrolizumab intravenously at a dose of 200 mg/body every three weeks as second-line therapy. Despite the immediate initiation of pembrolizumab, all metastatic sites were increased on CT after two courses of treatment, resulting in the discontinuation of pembrolizumab.

Clinical course of third-line treatment

**Figure 4:**
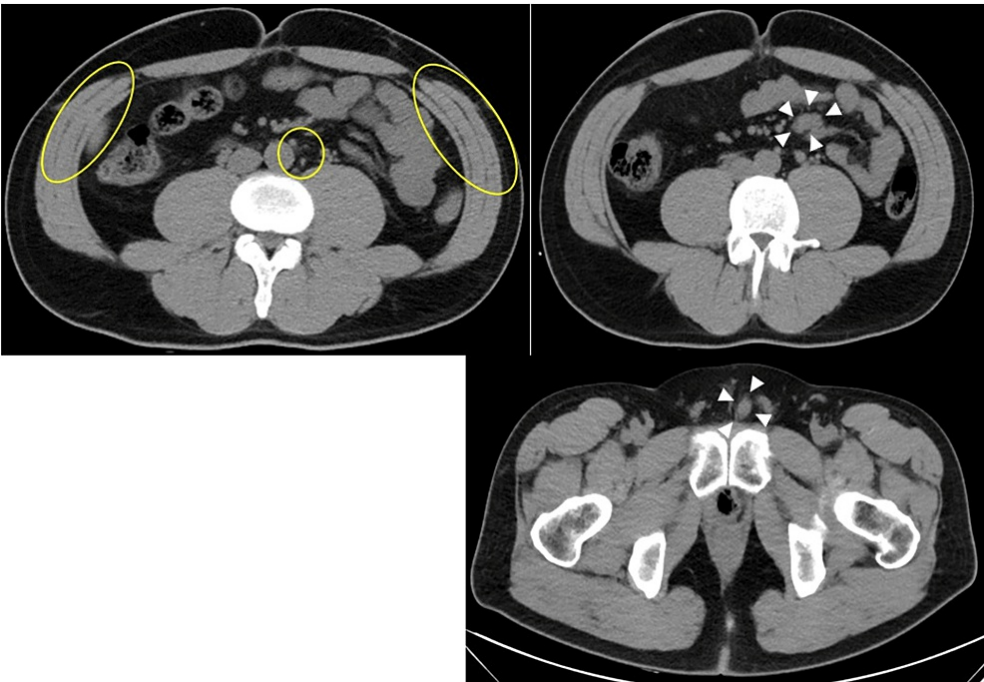
New lesions after the administration of enfortumab vedotin. Although computed tomography performed two months after the initiation of enfortumab vedotin (EV) exhibits metastases at other sites (white arrows), no re-growths of metastases that shrank with EV therapy were observed (yellow circles).

As the third-line treatment, EV 1.25 mg/kg was intravenously administered on days one, eight, and 15, and every 28 days thereafter. After two courses of EV, a follow-up CT scan exhibited a remarkable reduction of the tumor and almost complete disappearance from the metastatic site that existed at the time of recurrence. Two months later, the repeat CT scan showed metastases in other regions, and the patient was diagnosed with progressive disease without hydronephrosis. However, there was no regrowth of the metastasis, which was remarkably reduced by EV treatment (Figure 4). The patient continued to receive EV because there had been no rapid growth of new metastases 18 months after surgery. Although Cr and eGFR remained at 1.3-1.5 mg/dL and 40-45 mL/minute/1.73 m^2^, respectively, after starting pembrolizumab, PS maintained a good level of 0 during the follow-up period.

## Discussion

In 2019 and 2022, the U.S. Food and Drug Administration and the European Medicines Agency, respectively, approved EV for third-line treatment after platinum-based chemotherapy and ICI therapy [1,3,4]. EV is an antibody-drug complex, consisting of a human monoclonal antibody against Nectin-4 combined with a cytotoxic payload of the microtubule-disrupting compound monomethyl auristatin E (MMAE) [4]. EV binds to Nectin-4 and is transported to the intracellular lysosomes [5]. Subsequently, the linker is cleaved by the proteolytic enzymes, releasing MMAE into the cell, which inhibits tubulin polymerization, causing cell cycle arrest and apoptosis [5]. Nectin-4 is an intercellular adhesion factor that is known to be prominently expressed in the embryo, placenta, and skin, while it is also overexpressed in several malignant neoplasms, including urothelial and breast cancers [3]. Approximately 97% of urothelial carcinomas overexpress Nectin-4, which is recognized to play an important role in cancer cell proliferation, angiogenesis, and lymphatic invasion [4]. In total, approximately 70% of patients experienced treatment-related toxicity of any grade, and approximately 30% experienced grade 3-5 toxicity [6]. Among them, EV treatment was permanently discontinued in 10% of patients due to unacceptable toxicity [6]. The most common adverse event was peripheral sensory neuropathy, followed by dermatologic toxicity. Other adverse events such as ocular disturbances, impaired glucose control, and respiratory toxicity were also observed [3,6]. The patient in our case maintained good PS and had no EV-related adverse events during the follow-up period, suggesting that long-term treatment was feasible, and its effect may have been sustained.

In clinical practice, approximately one-third of patients with mUC could be treated with third-line therapy consisting of anticancer drugs only due to the progression of cancer or deterioration of the general condition [2]. In our previous study, 64.3% of the patients died from mUC during a median follow-up period of eight months [2]. The median OS and progression-free survival (PFS) in patients with mUC who received third-line chemotherapy were significantly longer than those who received no treatment [2]. These results suggest that third-line therapy may improve oncologic outcomes in patients with mUC [2]. In the EV-301 study, OS was significantly prolonged by approximately four months in patients with mUC treated with EV compared to those treated with chemotherapy [3]. Similarly, the PFS was significantly prolonged by approximately two months in patients with mUC treated with EV compared to those treated with chemotherapy [3]. On the other hand, the hazard ratio (HR) for OS was 0.69 in patients who had received one or two lines of prior therapy before the administration of EV, while the treatment effect was less robust in patients who received EV after the third line, with an HR of 0.88 [3]. PS is another factor contributing to prognosis and treatment efficacy [6]. This suggests that the limitations of the therapeutic effect of EV should be fully considered in patients with poor PS [6]. Although early metastasis was observed in this case, the patient had good PS and remained alive 18 months after surgery, suggesting that early EV administration may have been effective.

In this case, we did not perform adjuvant treatment as the patient declined it; however, it has been suggested that treatment with chemotherapy and/or ICIs may downregulate Nectin-4, and reduced Nectin-4 expression has been reported in patients with mUC receiving these therapies [4,5]. Although EV was successful once in this patient and there was no regrowth of the metastases, the reason for the new metastases at other sites was unclear. One possible reason is that cancer cells within a tumor may express different levels of Nectin-4. It is possible that the cancer cells expressing high levels of Nectin-4 shrank at the start of EV treatment, whereas high-grade cancer cells expressing low levels of Nectin-4 survived and metastasized to the new sites. These results suggest that oncological outcomes may improve when patients with mUC maintain good general health and receive third-line treatment with EV relatively early. Combination therapy of EV with various antitumor agents, such as pembrolizumab, cisplatin, or carboplatin, is currently being investigated [7]. If these trials demonstrate a therapeutic effect on mUC, EV could be administered earlier to patients with mUC, which may further improve oncologic outcomes.

## Conclusions

We report the case of a patient with mUC who responded to treatment with EV as a third-line therapy. Despite EV being the third-line treatment, multiple postoperative metastases were almost completely eliminated by EV. Conversely, the therapeutic effect of EV may differ depending on the site of metastasis as it did in this case. Therefore, it may be necessary to consider whether to continue EV treatment, switch to another treatment, or consider combination therapy such as that with EV and anticancer agents. Furthermore, it is necessary to consider the sequence of treatment to maximize the therapeutic effects of EV. In addition, it may be important to switch treatment regimens without delay so that EVs are used as early as possible.
